# Acceptability and safety of the menstrual cups among Iranian women: a cross-sectional study

**DOI:** 10.1186/s12905-021-01259-8

**Published:** 2021-03-13

**Authors:** Maryam Gharacheh, Fahimeh Ranjbar, Negar Hajinasab, Shima Haghani

**Affiliations:** 1 Nursing Care Research Center, School of Nursing and Midwifery, Iran University of Medical Sciences, Tehran, Iran; 2 Student Research Committee, Iran University of Medical Sciences, Tehran, Iran

**Keywords:** Menstruation, Menstrual hygiene, Menstrual management, Menstrual cup, Iran

## Abstract

**Background:**

Menstrual cup is one of the menstrual management products that is available throughout the world and can be effective in improving women's quality of life by empowering women in menstrual management. Although menstrual cups have recently entered the Iranian market, the use of this product is limited among women. The aim of this study was to determine the acceptability and safety of menstrual cups among Iranian women.

**Methods:**

This is a cross-sectional study conducted in 2020. Iranian women between the ages of 18 and 50 with regular menstrual periods who used the menstrual cup at least for three months were included. Participants were selected through continuous sampling, and they completed a web-based questionnaire including a demographic characteristics form as well as checklists on acceptability and safety through a link sent electronically via social media.

**Results:**

The mean score of the overall satisfaction with the cup was 6.54 ± 0.76, and the leakage had the lowest mean score among the satisfaction items (5.25 ± 1.63). About 83% of participants reported experiencing menstrual cup leakage. Among the reported health risks, the highest mean score was for vaginal pain during removal (23.9%). Most participants (83.9%) were familiar with the cup via social networks and 98.6% recommended this product to other women.

**Conclusions:**

The high level of acceptability and safety of the menstrual cup showed that this product is a suitable alternative for menstrual management in Iranian women. The results of the study help healthcare providers to learn more about the potential advantages and disadvantages of using cup and create trust in the menstrual cup use based on the results of local evidence-based research.

## Background

All over the world, women and girls use their own strategies for managing menstruation that vary from country to country, depending on personal preferences, available resources, economic status, indigenous traditions, cultural beliefs, and knowledge or education [1]. Lack of effective and affordable menstrual products makes the girls worried about leakage and unpleasant odor in school, leads to frequent school and workplace absenteeism, thereby affecting their health and education. Therefore, the number of programs that provide menstrual products for girls and women is increasing [2].

Menstrual cup is one of the menstrual management products available throughout the world [2]. This product is flexible and reusable, made of silicone and used to collect menstrual blood vaginally. These products can be purchased without a prescription and some models of cup can be used during intercourse. The willingness to use these cups is increasing, which is mainly due to the public's desire to use the eco-friendly period products [3, 4]. Menstrual cups can protect the environment by preventing from discarding millions of sanitary pads and tampons and improve the quality of life of women, especially athletes and working women, due to longer protection periods, leakage prevention, compatibility with activity and limiting the risk of toxic shock syndrome. This device can fill a major gap in menstrual hygiene products and women's reproductive health around the world, consequently empowering women in menstrual management [5, 6].

Evidence has shown the use of menstrual cup is not associated with negative effects on the vaginal flora. Limited cases of IUD dislodgement, severe pain or vaginal wounds, allergies, rashes, urinary tract problems (hydronephrosis) and toxic shock syndrome were reported after using a menstrual cup which required professional assistance to remove these products [2]. Preclinical assessments did not show any evidence that this product was toxic or mutagenic and no health risks were observed during the post-marketing study in the USA [3].

In general, the cup is preferred over other menstrual management methods because of its comfort, dryness, and less intense odor [7], and the major drivers for the acceptance of menstrual cup are increased comfort, independence, and mobility due to the reduced leakage compared to other methods [8]. In a study from the UK, 55% of participants indicated a desire to continue using the product due to less leakage during activity, environmentally-friendly design and its long-term cost-effectiveness [4]. However, discomfort in using the product, difficulty in removing the cup, or hygiene reasons, cramping, leakage and improper fit were the reasons for discontinuation in buying such products [3, 4].

Menstrual cup is available in 99 countries with 199 brands and price range of US$0·72-46·72; however, related information, which provides educational contents for women and girls, is usually not mentioned on websites. This product has received little attention that could be due to concerns about its use and cultural rejection or previous health warnings about tampons (for example, toxic shock syndrome). The number of people using menstrual cups is unknown. Therefore, it is not possible to compare the risk of toxic shock syndrome in using menstrual cups, tampons or vaginal diaphragms [2]. Although acceptability of this product has been reported among different cultures [5], the evidence suggests that women's views on the menstrual cups are related to socio-cultural factors [9]. Menstruation is still considered a taboo in some societies [5]. Given that the negative perceptions toward menstruation are a social construction, they can be challenged and changed. Positive experiences about the body and body functions effectively challenge negative social constructions. The use of alternative menstrual products may be a useful strategy in line with efforts to actively resist the social standards of beauty and femininity and menstrual etiquette. Many women have an androcentric view of menstruation that is characterized by stigma, negative attitudes, and sexual objectification related to menstruation [9].

Although menstrual cups are available and used around the world, based on the results of a systematic review and meta-analysis, there are few quality studies in this area and further research is needed on the safety and acceptability of these products. Information on leakage, acceptability and safety of menstrual cups is essential in order to make informed decisions and provide more comprehensive menstrual health education for women and girls. Further research can provide more information on the acceptability, cost-effectiveness, and environmental impacts of this product and should monitor the adverse consequences and evaluate best practice to shorten the familiarization phase for effective and safe use [2].

One-third of women in Iran are of childbearing age. Although menstrual cups with different brands have entered the Iranian market in recent years, the use of this product is very limited among Iranian women. Based on the knowledge of the researchers, no study in Iran has examined the acceptability and safety of this product. Given the importance of cultural and social issues in product adoption and to improve the strategies of women's menstrual management in Iran, this study was conducted to determine the acceptability and safety of menstrual cups among Iranian women.

## Methods

This cross-sectional study was conducted from June to September 2020. Iranian literate women between the ages of 18 and 50 with regular menstrual periods who used menstrual cups to manage menstruation for at least three consecutive cycles were included in the study. Endometriosis or fibroids, and silicone allergies were the exclusion criteria of the study.

The continuous sampling method was used to select the menstrual cup users in different provinces of Iran. Due to the limited number of users, samples were selected by snowball technique using virtual networks. The questionnaire link was sent to women electronically via social messengers to facilitate the collection of information. Google forms were used for designing and developing the web-based questionnaire. Using the formula to calculate the sample size with a confidence level of 95%, margin of error ± 1% (0.15) and using the standard deviation of 1.5 [10], the sample size for the chosen parameters was 400. Finally, a total of 515 participants were completed the questionnaire in current study.

To collect the data, a researcher-made tool was used, including a demographic and reproductive characteristics form (age, level of education, employment status, income, parity, gravidity, abortion and the contraception use) as well as a checklist on acceptability (leakage, comfort, insertion, removal, and overall satisfaction) and safety (vaginal irritation, vaginal pain when inserting and removing the cup, pelvic pain, allergies and rashes, physician-diagnosis of urinary tract infection, vaginitis, and toxic shock syndrome). The participants recorded acceptability on a seven-point Likert scale (from very bad to very good). Since there is no standard questionnaire, the study instrument was made after reviewing the literature and based on similar studies. In order to determine the validity of the instrument, the questionnaire was provided to a number of faculty members of the School of Nursing and Midwifery affiliated to Iran University of Medical Sciences and their opinions were applied. The Kuder–Richardson coefficient was used to determine the reliability of the instrument. Test–retest reliability coefficients for a 10 day interval were between 0.79 and 0.87 for leakage, comfort, insertion, removal and overall satisfaction.

The Ethics Committee of Iran University of medical sciences approved the project (Ethics code: IR.IUMS.REC.1399.290). The objectives of the study were presented to the participants and informed consent was obtained from all participants electronically. The participants were assured of data confidentiality and anonymity through assigning a code to each participant.

Descriptive statistics (such as central indicators and variance) and inferential statistics including independent T-test, analysis of variance, regression and Pearson correlation coefficient were used to analyze the data through the SPSS software version 16.

## Results

Among 600 participants that completed the survey, only 515 participants were eligible and were included in the study from July to September, 2020. The mean age of participants was 29.61 ± 5.65 and the mean age of their husbands was 34.29 ± 5.5. Most of the participants had a middle level of economic status (63.1%, 325). The provinces of Tehran (42.3%), Isfahan (7.6%) and Khorasan (7.2%) had the highest number of participants in the study. Most women had a bachelor's degree (49.5%, 225), were employed (48.2%, 248) and married (73.2%, 377). The demographic characteristics of the participants are presented in detail in Table 1.

**Table 1:** Demographic characteristics of participants (n = 515)

Category	n (%)	Mean (SD) minimum–maximum
**Age, years**		29.61 (5.65) 18–47
**Education level**		
Diploma	58 (11.3)	
Academic degree	457 (88.7)	
**Occupation**		
Housekeeping	172 (33.4)	
Jobs outside the home	248 (48.2)	
Work from home jobs	95 (18.4)	
**Marital status**		
Divorced	30 (5.8)	
Married	377 (73.2)	
Single	108 (21.0)	
**Duration of marriage; years**		6.89 (4.84) 1–27
**Husband’s age; years**		34.29 (5.50) 18–55
**Husband’s education level**		
High school	10 (2.6)	
Diploma	51 (13.1)	
Academic degree	327 (84.3)	
**Husband’s occupation**		
Employed	372 (96.4)	
Unemployed	14 (3.6)	
**Economic status**		
Low	22 (4.3)	
Middle	325 (63.1)	
High	168 (32.6)	
**Residence**		
Tehran	218 (42.3)	
Alborz	23 (4.5)	
Isfahan	39 (7.6)	
Fars	23 (4.5)	
Khusestan	33 (6.4)	
East and west Azarbayjan	18 (3.5)	
Gilan and Mazandaran	31(6.0)	
Khorasan (north, razavi and south)	37 (7.2)	
Kermanshah	16 (3.1)	
Kerman	15 (2.9)	
Markazi	10 (1.9)	
Other Provinces	52 (10.1)	

Most women were nulliparous (69.3%, 357) and used condom as a contraceptive method (31.7%, 163). The most common brand of cup used by the participants was Lunette Menstrual Cup (Lune Group Oy Ltd, Finland) (87.4%, 450). A total of 83.3% (429) reported experiencing menstrual cup leakage, 29.1% (125) had a history of at least one episode of leakage per cycle and 16.3% (70 people) had a history of ≥ 5 episodes of leakage per cycle. Most participants (49.1%, 253), changed their menstrual cup on average every 4–6 h (Table 2). Most participants (83.9%, 432) were familiar with this product via social networks and 98.6% (508) recommended this product to other women.

**Table 2:** Reproductive characteristics of participants (n = 515)

Category	n (%)	Mean (SD) Minimum–maximum
**Age at menarche**		13.26 (1.41) 8–18
**Number of pregnancies**		
0	338 (65.6)	
1	106 (20.6)	
≥ 2	71 (13.8)	
**Number of childbirth**		
0	357 (69.3)	
1	104 (20.2)	
≥ 2	54 (10.5)	
**Number of abortions**		
0	453 (88)	
1	49 (9.5)	
≥ 2	13 (2.5)	
**Number of alive children**		
0	356 (69.1)	
1	102 (19.8)	
≥ 2	57 (11.1)	
**Number of dead children**		
0	494 (95.9)	
1	15 (2.9)	
≥ 2	6 (1.2)	
**Current contraceptive methods**		
Condoms	163 (31.7)	
Withdrawal	117 (22.7)	
Withdrawal + condoms	105 (20.4)	
OCP	10 (1.9)	
Condoms + OCP	46 (8.9)	
Withdrawal + OCP	11 (2.1)	
IUD	10 (1.9)	
Other methods	16 (3.1)	
No methods	37 (7.2)	
**Cup's brand**		
Lunnet	450 (87.4)	
Meluna	40 (7.8)	
Nature	10 (1.9)	
Other	15 (2.9)	
**Leakage experience**		
No	86 (16.7)	
Yes	429 (83.3)	
**Number of episodes of leakage per cycle**		
1	125 (29.1)	
2	123 (28.7)	
3	70 (16.3)	
4	41 (9.6)	
≥ 5	70 (16.3)	
**Change frequency per cycle (h)**		
≤ 4	88 (17.1)	
4–6	253 (49.1)	
≥ 6	174 (33.8)	

The mean score of the overall satisfaction with the cup was 6.54 ± 0.76. The participants were more satisfied with comfort (6.69 ± 0.73) and less satisfied with leakage (5.25 ± 1.63) (Table 3). Among the safety items, the highest mean score was for vaginal pain during removal (23.9%, 123 patients) (Table 4).

**Table 3:** The acceptibility of the menstrual cup by participants (n = 515)

Satisfaction	Mean (SD)	Minimum-maximum
Leakage	5.25 (1.63)	1–7
Comfort	6.69 (0.73)	1–7
Insertion	6.04 (1.11)	1–7
Removal	6.21 (1.14)	1–7
Overall satisfaction	6.54 (0.76)	1–7

**Table 4:** The safety of the menstrual cups (n = 515)

Health risks	Category	n (%)
Vaginal irritation	No	467 (90.7)
	Yes	48 (9.3)
Vaginal pain during removal	No	392 (76.1)
	Yes	123 (23.9)
Subjective vaginal irritation	No	482 (93.6)
	Yes	33 (6.4)
Pelvic Pain	No	443 (86)
	Yes	72 (14)
Allergies and rashes	No	484 (94)
	Yes	31 (6)
Physician-diagnosed urinary tract infection	No	496 (96.3)
	Yes	19 (3.7)
Physician-diagnosed vaginitis	No	455 (88.3)
	Yes	60 (11.7)
Physician-diagnosed toxic shock syndrome	No	512 (99.4)
	Yes	3 (0.6)

Satisfaction had a significant inverse correlation with the participants' age, so that satisfaction decreased with increasing age (r = − 0.088, *P* = 0.047). Satisfaction was significantly higher [t(168.99) = 3.724, *P* = 0.001] in women who did not have pain during removal [t(168.99) = 3.724, *P* = 0.001], subjective vaginal pain [t(513) = 2.845, *P* = 0.005], pelvic pain [t(86.931) = 2.942, *P* = 0.004)], and allergies and rashes [t(32.693) = 2.256, *P* = 0.031] (Table 5).

**Table 5:** The relationship between demographic characteristics of participants and the safety items with the overall satisfaction with menstrual cups (n = 515)

	Overall satisfaction	
	Mean ± SD	Results
**Age, years**		*P* = 0.047^#^
**Education**		
Diploma	6.58 ± 0.81	0.685**
Academic degree	6.54 ± 0.76	
**Occupation**		
Housekeeping	6.57 ± 0.71	0.671*
Jobs outside the home	6.51 ± 0.82	
Work from home jobs	6.57 ± 0.70	
**Marital status**		
Divorced	6.66 ± 0.60	0.495*
Married	6.52 ± 0.79	
Single	6.59 ± 0.69	
**Duration of marriage; years**		*P* = 0.599^#^
**Economic status**		
Low	6.45 ± 0.96	0.734*
Middle	6.53 ± 0.79	
High	6.57 ± 0.69	
**Residence**		
Tehran	6.56 ± 0.78	0.612**
Other Provinces	6.52 ± 0.75	
**Vaginal irritation**		
No	6.56 ± 0.75	0.067**
Yes	6.35 ± 0.88	
**Vaginal pain during removal**		
No	6.61 ± 0.70	0.001**
Yes	6.32 ± 0.91	
**Subjective vaginal irritation**		
No	6.57 ± 0.72	0.005**
Yes	6.18 ± 1.15	
**Pelvic pain**		
No	6.59 ± 0.73	0.004**
Yes	6.26 ± 0.90	
**Allergies and rashes**		
No	6.57 ± 0.75	0.031**
Yes	6.19 ± 0.90	
**Physician-diagnosed urinary tract infection**		
No	6.55 ± 0.76	0.301**
Yes	6.36 ± 0.95	
**Physician-diagnosed vaginitis**		
No	6.55 ± 0.77	0.61**
Yes	6.50 ± 0.70	

*Oneway Anova

**Independent Samples Test

^#^Pearson Correlation

## Discussion

To the best of our knowledge, this is the first study to examine the acceptability and safety of menstrual cups in Iranian women. Participants in the current study were from all cities of Iran, which is one of the strengths of the study. Due to the limited evidence of menstrual cup use in Iran, the aim of this study was to provide evidence of the experience of using menstrual cup as a way to manage menstruation among Iranian women. Our results showed menstrual cup was a safe and acceptable mothod for most women and they had a positive experience using this product. Despite the high acceptance of this product around the world, the use of the cup is limited in Iranian women. Although Iran is one of the low and middle income countries (LMIC), access to sanitary toilets and tap water is provided almost everywhere, even in the rural or suburban areas. Therefore, it is easy to boil and disinfect the menstrual cup while preserving the privacy of the person. It seems that various cultural issues can affect the use of this product in Iran. Insufficient familiarity with the anatomy of the reproductive system due to poor puberty training in Iran and fear of touching and manipulating the reproductive organs can be an obstacle to the acceptance of this product in Iran. Most women in Iran have poor knowledge about menstrual hygiene [11] because talking about fertility and sexuality is a taboo, and girls are often not given enough information about puberty and menstruation at home or school due to cultural norms. This can lead to unhealthy behaviors and misconceptions regarding menstrual hygiene [12]. Furthermore, vaginal examination has been shown to cause discomfort, anxiety, and embarrassment for Iranian women [13], and perhaps due to inadequate training, the need to touch these organs during the insertion of the cup may lead to such feelings, discouraging them to use the product. Cultural norms about gender, sexuality, and female genitals, can make it difficult for a woman to touch these organs due to an unpleasant feeling. Women think that the female genitalia are a part of the body that are only related to sexuality and intimacy [14, 15]. Proper education of girls during puberty can reduce misconceptions about the reproductive system and turn menstruation, which is usually considered the worst experience of puberty by Iranian girls [12] into a positive experience. Therefore, encouraging women to use the menstrual cup for managing menstruation requires culture shaping and building. It has been shown that group training by peer groups can improve the use of menstrual cups [16], therefore, this should be considered in future interventions.

It seems that due to the taboo nature of extramarital affairs in Iran and its prohibition in Islamic teachings and the importance of maintaining virginity in unmarried women [17], this product is less accepted by adolescent girls, and unmarried women. Yet, almost one-fifth of those who completed the questionnaire were unmarried, which may indicate a difference in attitude toward breach of virginity among current consumers of the product compared to the general population in Iran. Despite all the cultural and religious restrictions, the acceptance of menstrual cups in our users shows the presence of a pool of potential customers in a population of Iranian women.

More than 90% of the participants stated that they would continue to use the cup and recommend it to others. These results were similar to those of other studies [3–5, 10, 18]. A systematic review and meta-analysis also showed that about 70% of the participants in 13 studies intended to continue use of this product.

In the present study, participants' overall satisfaction was high and similar to the study by Howard et al. in Canada [10]. The results of a clinical trial by Beksinska et al. in South Africa showed that compared to pads/tampons (usual products), the menstrual cup was rated better for comfort, quality, menstrual blood collection, appearance, and preference. These outcomes, along with possibility of continued use, recommending the product, and future purchase, increased the satisfaction with menstrual cups over time [18].

A large percentage of the participants complained about leakage, and among the items of satisfaction with the product, the item of leakage had the lowest score. However, the mean score of satisfaction with leakage (5.25 ± 1.63) was not very low and was similar to the study of Howard et al. (5.4 ± 1.4) [10]. Leakage was also a common item in the study of North and Oldham [3]. Conversely, complaints of leakage were 3–6% in the study of Kakani and Bhatt, which gradually decreased in the second and third cycles [7], and in the study of Madziyire Magure, and Madziwa, complaint about leakage was only 3% in the third cycle of use [19]. Complaints of leakage are usually reported in the early cycles of using the product and are more common in the first days of the menstrual cycle, which can be addressed by learning how to properly insert, and change the cup more frequently. By gaining experience in using the product, women can guess the right time to change the cup and minimize leakage. In the study of North and Oldham, despite the reports of leakage, women generally preferred cups over their previous methods of menstrual management [3].

Many complaints of leakage in the present study can be due to incorrect insertion of the cup. Poor puberty training in Iran and subsequent unfamiliarity with the anatomy of the reproductive system [12] can be the reasons for difficulty in inserting the cup. Most women purchase this product from online shopping websites or pharmacies and do not receive enough training on how to use the product. Open discussions and training at the initial visit and follow-up visits are very important in the acceptability of the menstrual cup [6]. The adoption of menstrual cup needs a familiarization phase during several menstrual cycles, and peer support can improve uptake rate [2]. The results of a study in South Africa also showed that nearly half of women (58%) reported that their initial difficulties in insertion had been reduced by repeated use. Similarly, the ease of removal had improved over time. The effect of practice in using female condoms has also been reported. Thus, women need to practice insertion in order to reduce discomfort and build self-confidence in correct insertion [18]. Accordingly, proper training in anatomy of the reproductive system through short educational videos is recommended.

In the present study, health risks such as vaginal irritation, subjective vaginal pain, pelvic pain, allergies and rashes, and toxic shock syndrome were very low (less than 15%) during the cup use. In addition, low rates of the health risks such as pain during removal, subjective vaginal pain, pelvic pain, and rashes and allergies, make the individuals satisfied with menstrual cup use which is a reasonable consequence. Vaginal pain during the removal of cup was the most common complaint. Difficulty in removal has also been reported in other studies [3, 7]. However, most women can overcome challenges such as insertion and removal by practice [18].

In studies from Kenya that compared the health outcomes of menstrual cups with sanitary pads, the prevalence of sexually transmitted diseases (STDs) and bacterial vaginosis in cup users was reported to be lower than sanitary pads [20, 21]. Also, no significant difference in urinary/vaginal symptoms diagnosed by a physician was reported between the two groups of menstrual cup and tampon [10]. Side effects of cup use such as infection, dryness, rash and allergies have been limited, so it seems that this product does not pose a health risk and is acceptable for many women without the need for measurement and fitting or other medical interventions [7]. Although not much is known about toxic shock syndrome, studies have shown that menstrual cups are not safer than tampons in terms of toxic shock syndrome [22], so similar to tampons, caution is recommended when using this product instead of a tampon, and training about timely removal of the cup is necessary.

This study has some limitations which need to be considered. Most participants were highly educated, employed and from the middle or upper economic class of the society. Thus, the generalizability of the results to groups with low socio-economic level can be difficult. Due to the limited number of people who use this product and the difficulty of accessing them, sampling was done non-randomly through social networks. In addition, the study was conducted as a single group whereas comparing the women’s satisfaction of cup with sanitary pads could enrich the study. It was not possible to compare the cup with a tampon, because tampons are not conventional menstrual products among Iranian women and are usually used only on certain days of a menstrual cycle, such as when going to the swimming pool or participating in sports. Since the participants in the present study were not novice cup users, it is natural that satisfaction with the product was higher than in studies in which women used the product for the first time.

Given that most participants complained about leakage, it is recommended to assess and compare the leakage on different menstrual days and assess the effect of education and training on reducing the leakage in future studies. As socio-cultural issues also play an important role in the acceptability of this product, it is recommended to conduct qualitative studies on women with racial and cultural diversity to gain a better understanding of the experience of using this product. The result of current study can be used as a basis for further interventional studies in Iran. Since the use of menstrual cups can lead to a positive menstrual experience in women, it can be expected that it will have other outcomes such as improvement in quality of life, and academic and career advancement, which require further studies in this area.

## Conclusions

The result showed that menstrual cup is a safe and acceptable method for menstrual management in Iranian women and can be proposed as an alternative and reusable menstrual hygiene product. Complaints of leakage and difficulty in removing the cup were the most common ones, which may be due to lack of training or unfamiliarity with the anatomy of the reproductive organs. Menstrual cup is not considered a common method of menstrual management in Iran and most of the participants were acquainted with this product through social networks, which could be due to cultural differences. This environmentally-friendly and cost effective alternative to pads/tampons can be a good option for women, especially during exercise time, travel or outdoor activities. Therefore, capacity building for women to get more familiar with the product is essential in future reproductive health policies. The results of the present study help reproductive healthcare professionals, including midwives, to learn more about the potential advantages and disadvantages of using cup and create trust in the cup use based on the results of local evidence-based research; therefore, accurate advice can be provided for girls and women on the efficacy of the menstrual hygiene products.

## Data Availability

The datasets used and analyzed during the present study are available from the corresponding author upon reasonable request.

## References

[1] Sommer M. Utilizing participatory and quantitative methods for effective menstrual-hygiene management related policy and planning. In: UNICEF-GPIA international conference: 2010; 2010.

[2] Van Eijk AM, Zulaika G, Lenchner M, Mason L, Sivakami M, Nyothach E, Unger H, Laserson K, Phillips-Howard PA (2019). Menstrual cup use, leakage, acceptability, safety, and availability: a systematic review and meta-analysis. Lancet Public Health.

[3] North BB, Oldham MJ (2011). Preclinical, clinical, and over-the-counter postmarketing experience with a new vaginal cup: menstrual collection. J Womens Health.

[4] Stewart K, Greer R, Powell M (2010). Women's experience of using the Mooncup. J Obstet Gynaecol.

[5] Shihata A, Brody S (2014). An innovative, reusable menstrual cup that enhances the quality of women’s lives during menstruation. J Adv Med Med Res.

[6] Tellier M, Hyttel M, Gad M. Pilot study report. 2012.

[7] Kakani C, Bhatt J (2017). Study of adaptability and efficacy of menstrual cup in managing. Study Adaptability efficacy of menstrual cup in managing.

[8] Hyttel M, Thomsen CF, Luff B, Storrusten H, Nyakato VN, Tellier M (2017). Drivers and challenges to use of menstrual cups among schoolgirls in rural Uganda: a qualitative study. Waterlines.

[9] Grose RG, Grabe S (2014). Sociocultural attitudes surrounding menstruation and alternative menstrual products: the explanatory role of self-objectification. Health Care Women Int.

[10] Howard C, Rose CL, Trouton K, Stamm H, Marentette D, Kirkpatrick N, Karalic S, Fernandez R, Paget J (2011). FLOW (finding lasting options for women): multicentre randomized controlled trial comparing tampons with menstrual cups. Can Fam Physician.

[11] Panjalipour S, Bostani Khalesi Z, Mirhaghjoo SN (2018). Iranian female adolescents’ reproductive health needs: a systematic review. IJWHR.

[12] Golchin NAH, Hamzehgardeshi Z, Fakhri M, Hamzehgardeshi L (2012). The experience of puberty in Iranian adolescent girls: a qualitative content analysis. BMC Public Health.

[13] Dabagh-Fekri S, Amiri-Farahani L, Amini L, Pezaro S (2020). A Survey of Iranian primiparous women’s perceptions of vaginal examination during labor. J Prim Care Community Health.

[14] Schooler D, Ward LM, Merriwether A, Caruthers AS (2005). Cycles of shame: menstrual shame, body shame, and sexual decision-making. J Sex Res.

[15] Sörensdotter R, Siwe K (2016). Touching the private parts: how gender and sexuality norms affect medical students’ first pelvic examination. Cult Health Sex.

[16] van Eijk AM, Laserson KF, Nyothach E, Oruko K, Omoto J, Mason L, Alexander K, Oduor C, Mohammed A, Eleveld A (2018). Use of menstrual cups among school girls: longitudinal observations nested in a randomised controlled feasibility study in rural western Kenya. Reproduct Health.

[17] Mehrolhassani MH, Yazdi-Feyzabadi V, Mirzaei S, Zolala F, Haghdoost A-A, Oroomiei N (2020). The concept of virginity from the perspective of Iranian adolescents: a qualitative study. BMC Public Health.

[18] Beksinska ME, Smit J, Greener R, Todd CS (2015). Lee M-lT, Maphumulo V, Hoffmann V: Acceptability and performance of the menstrual cup in South Africa: a randomized crossover trial comparing the menstrual cup to tampons or sanitary pads. J Womens Health.

[19] Madziyire MG, Magure TM, Madziwa CF (2018). Menstrual cups as a menstrual management method for low socioeconomic status women and girls in Zimbabwe: a pilot study. Women's Reprod Health.

[20] Phillips-Howard PA, Nyothach E, ter Kuile FO, Omoto J, Wang D, Zeh C, Onyango C, Mason L, Alexander KT, Odhiambo FO (2016). Menstrual cups and sanitary pads to reduce school attrition, and sexually transmitted and reproductive tract infections: a cluster randomised controlled feasibility study in rural western Kenya. BMJ Open.

[21] Juma J, Nyothach E, Laserson KF, Oduor C, Arita L, Ouma C, Oruko K, Omoto J, Mason L, Alexander KT (2017). Examining the safety of menstrual cups among rural primary school girls in western Kenya: observational studies nested in a randomised controlled feasibility study. BMJ Open.

[22] Nonfoux L, Chiaruzzi M, Badiou C, Baude J, Tristan A, Thioulouse J, Muller D, Prigent-Combaret C, Lina G (2018). Impact of currently marketed tampons and menstrual cups on Staphylococcus aureus growth and toxic shock syndrome toxin 1 production in vitro. Appl Environ Microbiol.

